# Editorial: Cognitive assessment in facilitating early detection of dementia

**DOI:** 10.3389/frdem.2024.1441582

**Published:** 2024-08-01

**Authors:** Pai-Yi Chiu, Chi-Cheng Yang, Mohammad H. Mahoor, Hsin-Te Chang

**Affiliations:** ^1^Department of Neurology, Show Chwan Memorial Hospital, Changhua City, Taiwan; ^2^Department of Applied Mathematics, Tunghai University, Taichung, Taiwan; ^3^Department of Psychology, College of Science, National Chengchi University, Taipei, Taiwan; ^4^Department of Electrical and Computer Engineering, University of Denver, Denver, CO, United States; ^5^Department of Psychology, College of Science, Chung Yuan Christian University, Taoyuan, Taiwan; ^6^Research Assistive Center, Show Chwan Memorial Hospital, Changhua City, Taiwan

**Keywords:** dementia, early detection, neuropsychological assessment, cognitive symptoms, mental processes

The rapid rise in global dementia prevalence poses severe challenges to health and social systems. Determining and early detecting cognitive decline and dementia risks are pressing issues for scientists. However, these issues are complicated by a lack of consensus in detecting, evaluating, and predicting pathological cognitive changes among patients. In this Research Topic, we gather multidisciplinary efforts to facilitate these processes, focusing on maximizing the effects of cognitive assessment on evaluating patients with dementia.

Ding et al. analyzed data from the Alzheimer's Disease Neuroimaging Initiative (ADNI), a large-scale, multi-center longitudinal study, using machine learning to demonstrate that the predictive power of performances on neuropsychological tests and neuroimaging results can be maximized. Littlejohn et al. used a dichotic listening paradigm, asking individuals with normal cognitive function, mild cognitive impairment (MCI), and dementia to choose between two consonant-vowel combination pairs. The study used a right-ear advantage in dichotic listening as an index of brain functioning. They found that when the task involved controlled attention to explicitly focus on one ear, the right-ear advantage decreased progressively among participants (i.e., control > MCI > dementia patients). Interestingly, the right-ear advantage was absent in MCI patients when the tasks did not require controlled attention. The authors suggest a compensatory mechanism in dichotic listening during the stages of MCI, proposing the task as an index for monitoring cognitive progression among dementia patients. Glenn et al. used a battery of eye-tracking and other computerized tasks to differentiate performances among cognitively normal individuals and patients with dementia of the Alzheimer's type. They found that the newly developed test battery had appropriate discriminative abilities and that performances correlated with those on standard cognitive screening tests. Ohno reviewed recent evidence and proposed accelerated long-term forgetting (ALF) as a marker for the earliest cognitive symptoms among patients with Alzheimer's disease. The association between amyloidosis and ALF suggests targeting mechanisms related to β secretase beta-site APP cleaving enzyme 1 (BACE1), which initiates amyloid-β production, as a potential means to halt cognitive decline.

These studies, along with other recent research (e.g., Bae et al., 2023; Ferretti et al., 2024), provide insights into the mechanisms and innovations in optimizing the procedures and models for detecting, assessing, and predicting cognitive decline and dementia. The studies in this Research Topic advance relatively undiscovered fields in dementia, including:

1. Developing innovative ways to early assess and monitor cognitive changes and characterize the neuropathological impact on cognitive functions in dementia patients.

2. Assessing cognitive changes in dementia patients with consideration for cost-effectiveness.

3. Incorporating knowledge of cognitive processes related to early pathological changes in developing therapies for dementia patients.

It is a precipitous time for initiating studies involving integrative approaches that combine several levels of analyses and modalities of information to further optimize the detection and assessment of dementia patients.

## Author contributions

P-YC: Writing – review & editing, Writing – original draft. MM: Writing – review & editing. C-CY: Writing – review & editing. H-TC: Writing – original draft, Writing – review & editing.
